# CLEC5A-Mediated Enhancement of the Inflammatory Response in Myeloid Cells Contributes to Influenza Virus Pathogenicity *In Vivo*

**DOI:** 10.1128/JVI.01813-16

**Published:** 2016-12-16

**Authors:** Ooiean Teng, Szu-Ting Chen, Tsui-Ling Hsu, Sin Fun Sia, Suzanne Cole, Sophie A. Valkenburg, Tzu-Yun Hsu, Jian Teddy Zheng, Wenwei Tu, Roberto Bruzzone, Joseph Sriyal Malik Peiris, Shie-Liang Hsieh, Hui-Ling Yen

**Affiliations:** a HKU-Pasteur Research Pole, School of Public Health, LKS Faculty of Medicine, The University of Hong Kong, Hong Kong SAR, China; b Centre of Influenza Research, School of Public Health, LKS Faculty of Medicine, The University of Hong Kong, Hong Kong SAR, China; c Institute of Clinical Medicine, National Yang-Ming University, Taipei, Taiwan; d Genomics Research Center, Academia Sinica, Taipei, Taiwan; e Department of Pediatrics and Adolescent Medicine, LKS Faculty of Medicine, The University of Hong Kong, Hong Kong SAR, China; f Department of Medical Research, Taipei Veterans General Hospital, Taipei, Taiwan; University of Iowa

**Keywords:** C-type lectins, CLEC5A, spleen tyrosine kinase (Syk), influenza virus, macrophages

## Abstract

Human infections with influenza viruses exhibit mild to severe clinical outcomes as a result of complex virus-host interactions. Induction of inflammatory mediators via pattern recognition receptors may dictate subsequent host responses for pathogen clearance and tissue damage. We identified that human C-type lectin domain family 5 member A (CLEC5A) interacts with the hemagglutinin protein of influenza viruses expressed on lentiviral pseudoparticles through lectin screening. Silencing CLEC5A gene expression, blocking influenza-CLEC5A interactions with anti-CLEC5A antibodies, or dampening CLEC5A-mediated signaling using a spleen tyrosine kinase inhibitor consistently reduced the levels of proinflammatory cytokines produced by human macrophages without affecting the replication of influenza A viruses of different subtypes. Infection of bone marrow-derived macrophages from CLEC5A-deficient mice showed reduced levels of tumor necrosis factor alpha (TNF-α) and IP-10 but elevated alpha interferon (IFN-α) compared to those of wild-type mice. The heightened type I IFN response in the macrophages of CLEC5A-deficient mice was associated with upregulated TLR3 mRNA after treatment with double-stranded RNA. Upon lethal challenges with a recombinant H5N1 virus, CLEC5A-deficient mice showed reduced levels of proinflammatory cytokines, decreased immune cell infiltration in the lungs, and improved survival compared to the wild-type mice, despite comparable viral loads noted throughout the course of infection. The survival difference was more prominent at a lower dose of inoculum. Our results suggest that CLEC5A-mediated enhancement of the inflammatory response in myeloid cells contributes to influenza pathogenicity *in vivo* and may be considered a therapeutic target in combination with effective antivirals. Well-orchestrated host responses together with effective viral clearance are critical for optimal clinical outcome after influenza infections.

**IMPORTANCE** Multiple pattern recognition receptors work in synergy to sense viral RNA or proteins synthesized during influenza replication and mediate host responses for viral control. Well-orchestrated host responses may help to maintain the inflammatory response to minimize tissue damage while inducing an effective adaptive immune response for viral clearance. We identified that CLEC5A, a C-type lectin receptor which has previously been reported to mediate flavivirus-induced inflammatory responses, enhanced induction of proinflammatory cytokines and chemokines in myeloid cells after influenza infections. CLEC5A-deficient mice infected with influenza virus showed reduced inflammation in the lungs and improved survival compared to that of the wild-type mice despite comparable viral loads. The survival difference was more prominent at a lower dose of inoculum. Collectively, our results suggest that dampening CLEC5A-mediated inflammatory responses in myeloid cells reduces immunopathogenesis after influenza infections.

## INTRODUCTION

Influenza viruses impact human health through annual epidemics, intermittent pandemics, and sporadic zoonotic infections. Infection may lead to self-limited or severe clinical symptoms as a result of complex virus-host interactions (1–5). Activation of pattern recognition receptors (PRRs) that rapidly respond to invading pathogens or damage-associated molecular patterns (DAMP) through induction of inflammatory mediators may dictate the subsequent adaptive immune response for pathogen clearance and clinical outcome. Various intracellular PRRs, including the Toll-like receptors (TLR) 3, 7, and 10 (6–9), retinoic acid-inducible gene I (RIG-I) (10), or Nod-like receptors NLRP3 and NOD2 (11–13), sense viral RNA or proteins produced during influenza virus replication, leading to induction of an inflammatory response followed by activation of CD4^+^ or CD8^+^ T cells (5, 14). Poorly coordinated host responses may pave the way for severe clinical outcomes characterized by elevated inflammatory responses, increased leukocyte infiltration, and pulmonary tissue injury (15–19).

Signaling through C-type lectin receptors (CLRs) may further modulate host defense against infections by different microbes (20). The C-type lectin superfamily includes soluble or integral proteins containing the characteristic C-type lectin-like domain (CTLD) that binds to carbohydrates, lipids, or proteins (21). The integral CLRs on myeloid cells can signal through the immunoreceptor tyrosine-based activation motif (ITAM) or immunoreceptor tyrosine-based inhibition motif (ITIM) signaling motifs either directly or in association with other adaptor proteins (21, 22) and trigger a plethora of myeloid cell responses, such as phagocytosis (23), priming cytotoxic T lymphocytes (24), respiratory burst (25), or production of proinflammatory cytokines (26, 27). Furthermore, CLR signaling may antagonize or synergize the signals from other PRRs to regulate the host immune response (21, 22).

Multiple soluble C-type lectins, such as mannose-binding lectin (MBL), surfactant protein D (SP-D), and collectin kidney 1, possess neutralizing activity against influenza virus during infection (28–30). Galectin-1 of the S-type lectin family and the serum amyloid P of the pentraxin family have also been shown to ameliorate influenza infectivity by inhibiting hemagglutinin (HA) activity (31, 32). In addition, CLRs expressed on myeloid cells, including DC-SIGN, DC-SIGNR, macrophage mannose receptor (MMR), and macrophage galactose-type lectin (MGL), can mediate influenza virus internalization in a sialic acid-independent manner (33–36). However, it is not known if influenza infections trigger signaling via myeloid CLRs that further modulate the host immune response. Here, we identify that the spleen tyrosine kinase-coupled C-type lectin domain family 5 member A (CLEC5A), which has previously been reported to mediate flavivirus-induced inflammatory response (26, 27, 37), enhanced induction of proinflammatory cytokines and chemokines in myeloid cells after influenza A virus infection. Blockade of CLEC5A-mediated signaling attenuated influenza virus-induced inflammatory responses in macrophages without affecting viral replication. With reduced cytokines but comparable viral loads in mouse lungs, the CLEC5A knockout (CLEC5A^−/−^) mice were protected after lethal influenza challenge compared to C57BL/6 wild-type (WT) mice, although a more prominent difference was observed when they were challenged with a lower inoculum. This suggested that dampening CLEC5A-mediated inflammatory responses in the myeloid cells is able to alleviate influenza pathogenicity *in vivo*. Our results highlight the significance of viral load control and a well-orchestrated host response in influenza pathogenesis.

## RESULTS

### Binding of influenza HA-expressing pseudotyped lentiviral particles to human soluble CLEC5A.

**FIG 1:**
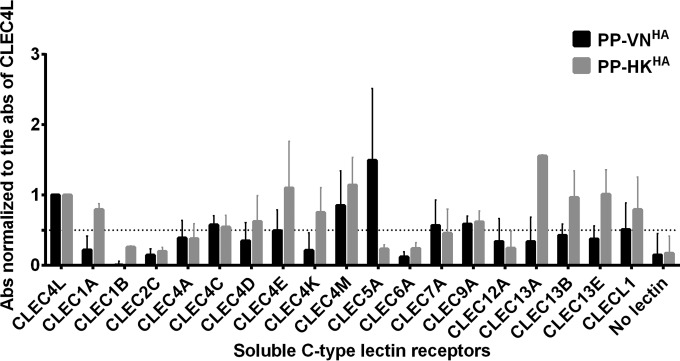
Identification of CLEC5A as potential PRR for influenza virus. The relative binding intensity of the pseudoparticles expressing influenza HA derived from H5N1 or H1N1 viruses to a panel of soluble human CLRs is shown. Briefly, 0.5 μg of soluble human CLRs was coated on the plate, followed by incubation with the HA-expressing pseudoparticles. The bound pseudoparticles were detected by anti-H5 or anti-H1 antibodies. The absorbance (Abs) obtained was normalized to the absorbance of CLEC4L. The dotted line represents 50% binding signal compared to that of CLEC4L. The data shown are the means ± standard deviations (SD) from 8 replicates from 4 independent experiments.

The hemagglutinin (HA) glycoproteins are abundantly present on the surface of influenza virions and have been reported to interact with different CLRs to facilitate viral entry through glycans synthesized during posttranslational modifications (33–36). Differential inflammatory cytokine responses have been reported after infections with H5N1 and H1N1 influenza viruses (16, 38, 39). To investigate the potential interactions between influenza virus and human CLRs, pseudotyped lentiviral particles with surface expression of influenza HA proteins derived from an H5N1 isolate, A/Vietnam/1203/04 (PP-VN^HA^), or a seasonal H1N1 isolate, A/Hong Kong/54/98 (PP-HK^HA^), were coincubated with a panel of soluble recombinant human CLR-Fc fusion proteins precoated on 96-well plates. The functions of the HA proteins expressed by PP-VN^HA^ and PP-HK^HA^ resemble the biological functions of those presented by influenza viruses with hemagglutination activity to turkey red blood cells, and they mediate entry and the transduction of renilla luciferase reporter in MDCK cells (data not shown). Pseudoparticles without surface glycoprotein expression were coincubated with soluble recombinant human CLR-Fc fusion proteins as negative controls to determine the background binding signals. The binding intensity of the HA-expressing pseudoparticles to each CLR was normalized to the binding intensity to CLEC4L (DC-SIGN), which is known to interact with influenza HA (33) (Fig. 1). CLEC4M (DC-SIGNR), CLEC7A, CLEC4C, CLEC9A, and CLEC5A gave more than 50% binding signal relative to that of CLEC4L for PP-VN^HA,NA^. The detection of a positive signal indicates that there is either a direct interaction between the soluble CLR and the HA-expressing pseudoparticles or that there is an indirect mediator bound to both the soluble CLR and the HA-expressing pseudoparticles. For example, DC-SIGN is known to recognize the high-mannose N-linked glycans present on influenza HA glycoprotein (33). CLEC5A was selected for further investigation, as it gave the highest binding signal to the PP-VN^HA^ virus expressing H5 HA protein. CLEC5A activation by dengue virus (DV) or Japanese encephalitis virus (JEV) induces DAP12 phosphorylation and mediates induction of proinflammatory cytokines (tumor necrosis factor alpha [TNF-α] and interleukin-6 [IL-6]) and chemokines (IP-10, MIP-1α, monocyte chemoattractant protein 1 [MCP-1], and IL-8) (26, 37, 40). Since hyperinduction of proinflammatory cytokines is a signature for H5N1 pathogenesis (16), we hypothesized that CLEC5A is involved in regulating the host inflammatory response upon influenza virus infection.

### CLEC5A enhanced proinflammatory response in human PBMC-derived macrophages after influenza virus infection.

To study the CLEC5A-mediated host response, human peripheral blood mononuclear cells (PBMCs) obtained from four donors were each differentiated into macrophages and dendritic cells, and CLEC5A surface expression was monitored. High levels of CLEC5A were detected on CD14^+^/major histocompatibility complex class II-positive (MHC-II^+^) macrophages differentiated with macrophage colony-stimulating factor (M-CSF) (M-Mϕ) (72.9% ± 8.9%) or granulocyte-macrophage colony-stimulating factor (GM-CSF) (GM-Mϕ) (94.8% ± 4.9%) but not on those differentiated with autologous serum (17.2% ± 11.4%) or on the CD14^−^/MHC-II^+^ dendritic cells differentiated with GM-CSF and IL-4 (11.9% ± 9.0%) (data not shown). Short interfering RNA (siRNA)-mediated gene silencing was applied to knock down CLEC5A expression in M-Mϕ and GM-Mϕ. Among the primary macrophages transfected with nontargeting control siRNA at 72 h posttransfection, 89.6% of cells expressed CLEC5A on the cell surface; in comparison, 71.1% of the primary macrophages transfected with the siRNA targeting human CLEC5A expressed CLEC5A. Due to the inefficient siRNA knockdown efficiency in primary macrophages, we proceeded to cell sorting after siRNA knockdown to yield CLEC5A^+^ and CLEC5A^−^ populations as described in Materials and Methods. Specifically, at 72 h after siRNA knockdown followed by negative cell sorting, 79.4% of the M-Mϕ transfected with nontargeting control siRNA (siNT) and 15.6% of the M-Mϕ transfected with CLCE5A-specific siRNA (siCLEC5A) showed surface expression of CLEC5A. We observed that the surface expression of CLEC5A was lowest at 72 h posttransfection and continued to remain low at 96 h posttransfection (data not shown). As such, infections with influenza viruses were performed at 72 h posttransfection with samples collected 24 h later (96 h posttransfection).

**FIG 2:**
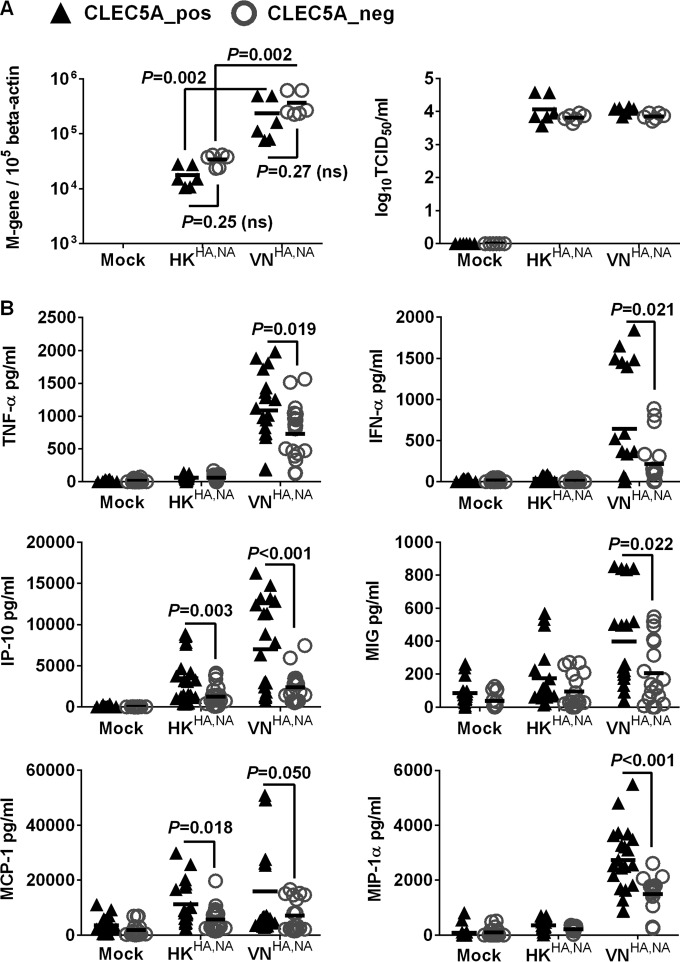
CLEC5A mediates enhanced proinflammatory cytokine and chemokine induction in human M-Mϕ after influenza virus infection. M-Mϕ differentiated from the PBMC of 8 independent donors were transfected with CLEC5A gene-specific or nontargeting siRNA, followed by cell sorting to obtain the CLEC5A-positive (CLEC5A_pos) and CLEC5A-negative (CLEC5A_neg) populations. The macrophages were then infected with HK^HA,NA^ or VN^HA,NA^ recombinant viruses at an MOI of 2 for 24 h to determine viral M gene copy numbers in infected M-Mϕ and viral titers in culture supernatants (log_10_ TCID_50_/ml in MDCK cells) (A) and proinflammatory cytokines and chemokines in culture supernatant (means ± SD, in pg/ml) (B). *P* values from Mann-Whitney tests are shown.

**FIG 3:**
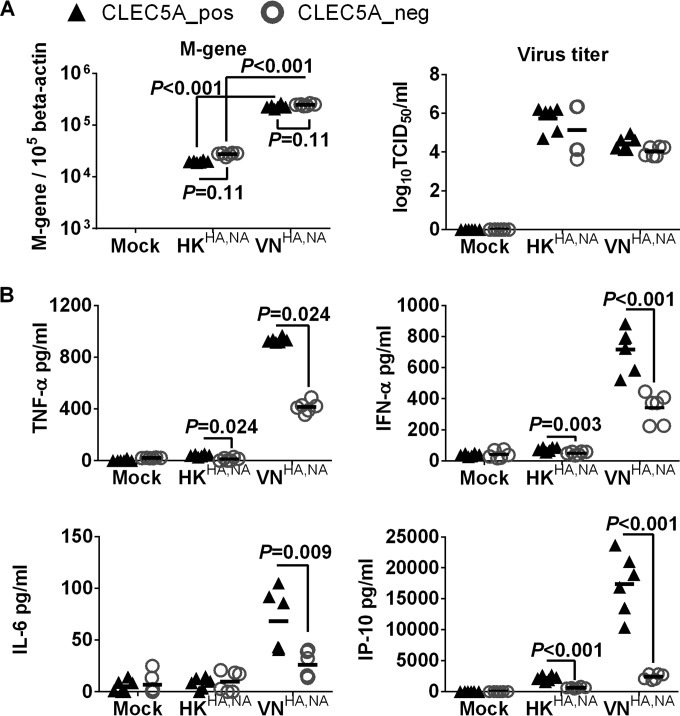
CLEC5A mediates enhanced proinflammatory cytokine and chemokine induction in human GM-Mϕ after influenza virus infection. GM-Mϕ differentiated from the PBMC of 2 independent donors were transfected with CLEC5A gene-specific or nontargeting siRNA, followed by cell sorting to obtain the CLEC5A-positive (CLEC5A_pos) and CLEC5A-negative (CLEC5A_neg) populations. The macrophages were then infected with HK^HA,NA^ or VN^HA,NA^ recombinant viruses at an MOI of 2 for 24 h to determine viral M gene copy numbers in infected GM-Mϕ and viral titers in culture supernatant (log_10_ TCID_50_/ml in MDCK cells) (A) and proinflammatory cytokines and chemokines in culture supernatants (means ± SD, in pg/ml) (B). *P* values from Mann-Whitney tests are shown.

Previous studies from our laboratory have demonstrated differential proinflammatory cytokine responses in human macrophages after infections with A/Vietnam/1203/04 (H5N1) and A/HK/54/98 (H1N1) influenza viruses (38, 39). We hypothesized that such differential cytokine responses are induced through interactions between influenza surface glycoprotein and CLEC5A. The CLEC5A^+^ and CLEC5A^−^ M-Mϕ and GM-Mϕ were infected with recombinant influenza viruses with identical internal genes derived from the A/PR/8/34 virus but with different surface HA and NA glycoproteins derived from the A/Hong Kong/54/98 (H1N1) (denoted HK^HA,NA^) and A/Vietnam/1203/04 (H5N1) (denoted VN^HA,NA^) viruses based on the hypothesis that the interaction between CLR and influenza virus was mediated via viral surface glycoproteins. After infection with the VN^HA,NA^ virus, the CLEC5A^−^ and CLEC5A^+^ cells showed comparable M gene copies, suggesting that CLEC5A does not affect viral entry or replication in M-Mϕ (Fig. 2A) or GM-Mϕ (Fig. 3A). However, significantly lower levels of cytokines (TNF-α, alpha interferon [IFN-α], and IL-6) and chemokines (IP-10, MIP-1α, MCP-1, and MIG) were detected in the CLEC5A^−^ M-Mϕ (Fig. 2B) or GM-Mϕ (Fig. 3B) than in the CLEC5A^+^ cells (*P* values of <0.05 by Mann-Whitney test).

Compared to the H5N1 VN^HA,NA^ virus, the H1N1 HK^HA,NA^ virus showed lower infectivity in the M-Mϕ (Fig. 2A) and GM-Mϕ (Fig. 3A) and induced lower levels of proinflammatory cytokines in M-Mϕ (Fig. 2B) and GM-Mϕ (Fig. 3B). Infection of HK^HA,NA^ virus in CLEC5A^−^ and CLEC5A^+^ cells showed comparable M gene copies in M-Mϕ (Fig. 2A) or GM-Mϕ (Fig. 3A); however, reduced levels of IP-10, MCP-1, TNF-α, and IFN-α were observed from the CLEC5A^−^ M-Mϕ (Fig. 2B) or GM-Mϕ (Fig. 3B) than the CLEC5A^+^ cells, although the level of reduction was not as obvious as that observed for the VN^HA,NA^ virus. IL-4, IL-10, IL-12p70, and transforming growth factor beta (TGF-β) were under the detection limit. Overall, the results suggest that CLEC5A mediates enhanced inflammatory responses in M-Mϕ and GM-Mϕ after infection with the recombinant H5N1 or H1N1 influenza viruss.

### Enhanced inflammatory response mediated by CLEC5A is subtype independent.

**FIG 4:**
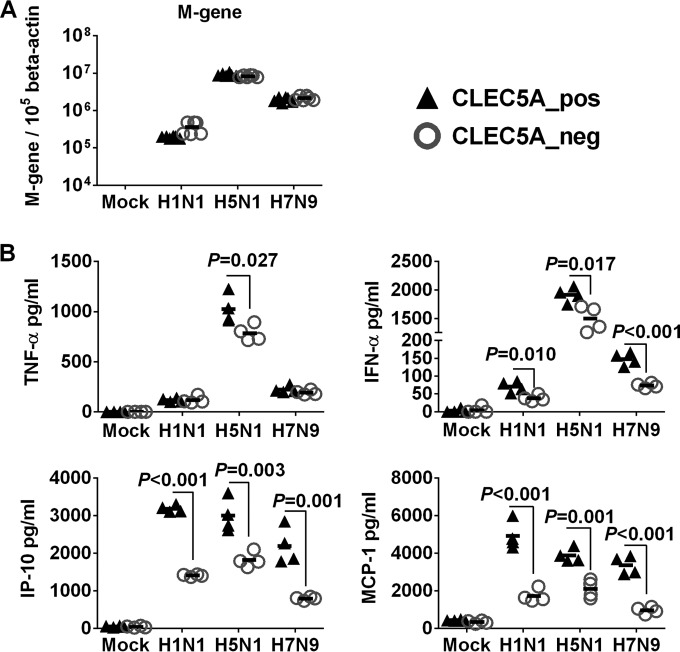
CLEC5A-mediated proinflammatory response in human M-Mϕ after infections with influenza viruses of H1N1, H5N1, and H7N9 subtypes. Human M-Mϕ differentiated from PBMC of 2 independent donors were infected with A/Hong Kong/54/98 (H1N1), A/Vietnam/1203/04 (H5N1), or A/Shanghai/2/13 (H7N9) influenza viruses at an MOI of 2 for 24 h to determine viral M gene copy numbers in infected M-Mϕ (A) and proinflammatory cytokines and chemokines in culture supernatants (means ± SD, in pg/ml) (B). *P* values from Mann-Whitney tests are shown.

We further applied wild-type A/Hong Kong/54/98 (H1N1), A/Vietnam/1203/04 (H5N1), and A/Shanghai/2/13 (H7N9) viruses to assess if the enhanced inflammatory response mediated by CLEC5A would be affected by different influenza strains that differed by the gene constellations in all eight-gene segments. The H5N1 virus showed the highest infectivity, followed by the H7N9 and H1N1 viruses in human M-Mϕ (Fig. 4A). H5N1 virus induced higher levels of TNF-α and IFN-α in the infected M-Mϕ (Fig. 4B), but the levels of chemokines (IP-10 and MCP-1) were comparable after infection with H1N1, H5N1, or H7N9 viruses. Despite possessing different infectivity, all three viruses showed comparable infectivity in CLEC5A^−^ and CLEC5A^+^ populations (Fig. 4A), further supporting that the CLEC5A cells did not affect viral entry or replication in M-Mϕ. Silencing of CLEC5A significantly reduced the level of TNF-α in H5N1-infected M-Mϕ and the levels of IFN-α, IP-10, and MCP-1 in M-Mϕ infected by H5N1, H7N9, or H1N1 viruses (Fig. 4B). Overall, these results suggest that CLEC5A mediates proinflammatory cytokine and chemokine induction in human PBMC-derived macrophages independent of the influenza virus subtypes.

### Blocking CLEC5A-mediated signaling reduced inflammatory response in M-Mϕ after influenza infection.

**FIG 5:**
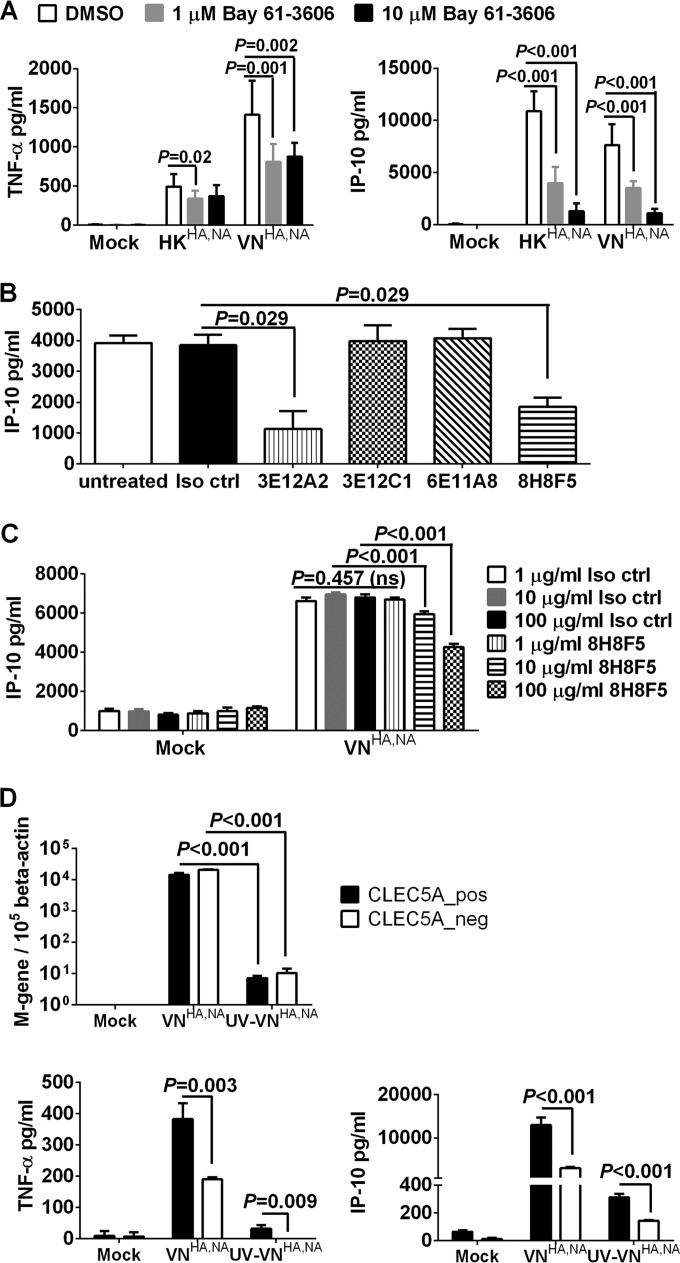
Blocking CLEC5A-mediated signaling with Syk inhibitor (Bay 61-3606) and CLEC5A antagonizing antibodies reduced inflammatory response in human M-Mϕ after influenza virus infection. (A) Human M-Mϕ differentiated from the PBMC of 3 independent donors were treated with dimethyl sulfoxide or the Syk inhibitor Bay 61-3606 prior to and throughout the course of infection with HK^HA,NA^ or VN^HA,NA^ virus at an MOI of 2 to determine the TNF-α and IP-10 concentrations (means ± SD, in pg/ml) from culture supernatants at 24 h postinfection. (B) Human M-Mϕ differentiated from the PBMC of 4 independent donors were incubated with 1 μg/ml of murine IgG1 isotype control antibody (Iso ctrl) or clones of anti-human CLEC5A antibodies, which were previously shown to possess antagonizing activity against DV or JEV infections, prior to and throughout the course of infection with VN^HA,NA^ virus at an MOI of 2 to determine the level of IP-10 (means ± SD, in pg/ml) from culture supernatants at 24 h postinfection. (C) Human M-Mϕ differentiated from the PBMC of 4 independent donors were incubated with increasing concentrations of murine IgG1 isotype control antibody or the anti-CLEC5A antibody (clone 8H8F5) prior to and throughout the course of infection with VN^HA,NA^ virus at an MOI of 2 to determine the level of IP-10 (means ± SD, in pg/ml) from culture supernatants at 24 h postinfection. ns, not significant. (D) M-Mϕ differentiated from the PBMC of 2 independent donors were transfected with CLEC5A gene-specific or nontargeting siRNA followed by cell sorting to obtain the CLEC5A-positive (CLEC5A_pos) and CLEC5A-negative (CLEC5A_neg) populations. M-Mϕ were infected with VN^HA,NA^ or UV-inactivated VN^HA,NA^ (UV-VN^HA,NA^) at an MOI of 2. Viral M gene copy numbers and levels of TNF-α and IP-10 (means ± SD, in pg/ml) in culture supernatant were determined at 24 h postinfection. *P* values from Mann-Whitney tests are shown.

CLEC5A mediates inflammatory responses through association with the ITAM-containing DAP12 or the YINM-containing DAP10 adaptor proteins, followed by spleen tyrosine kinase (Syk) or phosphoinositide 3-kinase (PI3K)-mediated signaling pathways (22, 26, 27, 40, 41). Since Syk-mediated signaling was critical for CLEC5A-induced lethal shock in mice (41) and inflammasome activation in DV pathogenesis (27), we asked if Syk-mediated signaling is involved in the release of proinflammatory cytokines after influenza infection. To address this question, M-Mϕ were incubated with the selective Syk inhibitor Bay 61-3606 (42) 1 h prior to and throughout the course of infection with HK^HA,NA^ or VN^HA,NA^ viruses. The concentrations of IP-10 and TNF-α were reduced under increasing concentrations of Bay 61-3606 (Fig. 5A), while the viral M gene copies remained unaffected (data not shown). We further asked if blockade of the influenza virus-CLEC5A interaction is able to reduce proinflammatory cytokine induction in human macrophages. Panels of antagonizing mouse anti-human CLEC5A antibodies (clones 3E12A2, 3E12C1, 6E11A8, and 8H8F5) previously shown to block DV- or JEV-induced proinflammatory cytokine releases from human macrophages (26, 27, 37) were preincubated with M-Mϕ 1 h prior to and throughout the course of infection with VN^HA,NA^ virus. We found that clones 8H8F5 and 3E12A2 inhibit IP-10 secretion from M-Mϕ after VN^HA,NA^ virus infection (Fig. 5B) in a dose-dependent manner (Fig. 5C). The anti-CLEC5A blocking antibodies had no impact on influenza replication, as the M gene copies detected in the M-Mϕ were comparable to those of the isotype control-treated macrophages. Overall, these observations further support the role of CLEC5A in mediating induction of proinflammatory cytokines in human macrophages after influenza infection.

To further examine if binding of influenza virus to CLEC5A expressed on the cell surface is sufficient to trigger the proinflammatory cytokine or chemokine induction, CLEC5A^−^ and CLEC5A^+^ M-CSF macrophages were infected with live or UV-inactivated VN^HA,NA^ viruses (UV-VN^HA,NA^) at a multiplicity of infection (MOI) of 2. The UV-VN^HA,NA^ virus still possessed hemagglutination activity to turkey red blood cells (data not shown) but lacked infectivity, as the M gene copy numbers remained low at 24 h postinfection compared to those of live VN^HA,NA^ (Fig. 5D). Minimal induction of cytokines was observed in CLEC5A^−^ and CLEC5A^+^ M-Mϕ after infection with the UV-VN^HA,NA^ virus (Fig. 5D). Similar results were observed when the MOI was increased from 2 to 20 (data not shown). This observation is in accord with our previous observation that UV-inactivated DV is less potent than live DV to activate CLEC5A (26), and active viral replication is required for the CLEC5A-mediated enhancement of the inflammatory response in M-Mϕ.

### Influenza infections in bone marrow-derived macrophages of CLEC5A^−/−^ mice showed reduced levels of TNF-α and IP-10 but increased IFN-α compared WT mice.

**FIG 6:**
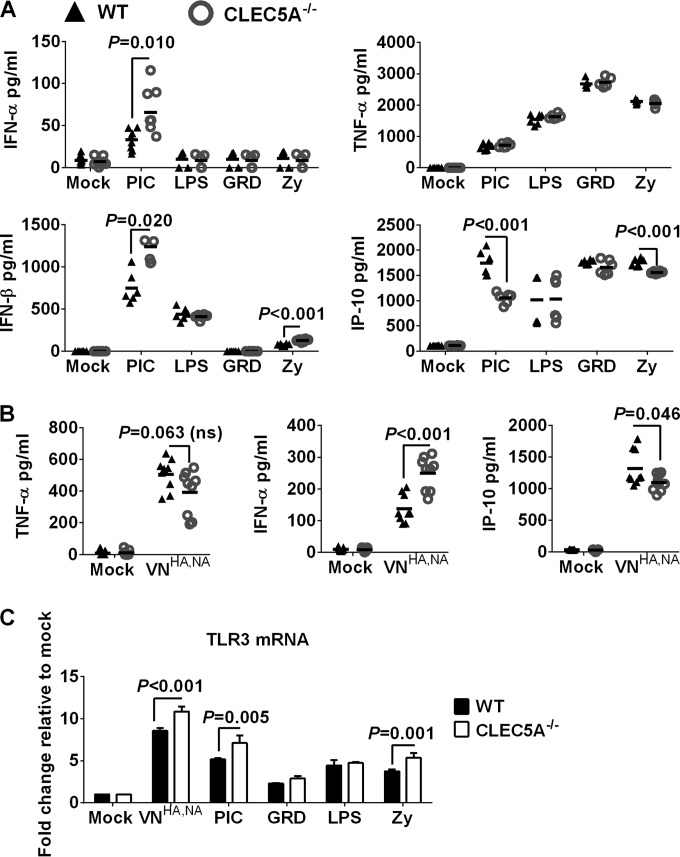
(A) Inflammatory response in BMM derived from CLEC5A^−/−^ or WT mice after incubation with TLR agonists or after influenza infection. BMM derived from CLEC5A^−/−^ or WT mice were treated with gardiquimod (GRD; 10 μg/ml), lipopolysaccharide (LPS; 100 ng/ml), poly(I·C) (PIC; 50 μg/ml), or zymosan (Zy; 10 μg/ml) for 24 h to determine the level of cytokines and chemokines in the supernatant (means ± SD, in pg/ml, derived from 6 replicates of BMM obtained from 2 mice). (B) BMM derived from CLEC5A^−/−^ or WT mice were infected with VN^HA,NA^ at an MOI of 2 for 24 h to determine the levels of cytokines and chemokines in the supernatant (means ± SD, in pg/ml, derived from 9 replicates of BMM obtained from 3 mice). (C) Copy number of TLR3 mRNA in BMM after activation with TLR agonist or influenza infection at an MOI of 2 for 24 h (means ± SD from 4 replicates of BMM obtained from 2 mice). *P* values from Mann-Whitney tests are shown.

CLEC5A^−/−^ mice with deletion of exons 3 to 5 of the murine *Clec5a* genomic DNA (37) and the genetically matched WT mice were applied to evaluate the impact of CLEC5A on influenza pathogenesis. We first characterized the inflammatory response of bone marrow-derived macrophages (BMM) from CLEC5A^−/−^ or WT mice after treatment with zymosan (Zy; TLR2 agonist), poly(I·C) (PIC; TLR3 agonist), lipopolysaccharide (LPS; TLR4 agonist), or gardiquimod (GRD; TLR7 agonist). The levels of TNF-α were comparable between BMM derived from CLEC5A^−/−^ and WT mice after treatment with PIC, LPS, GRD, or Zy (Fig. 6A). However, BMM from CLEC5A^−/−^ mice showed an increased type I interferon response compared to that of the WT mice after PIC treatment (Fig. 6A). In addition, reduced IP-10 was detected from the macrophages derived from the CLEC5A^−/−^ mice after treatment with PIC or Zy.

BMM derived from CLEC5A^−/−^ or WT mice were infected with VN^HA,NA^ virus at an MOI of 2, and the levels of cytokines and chemokines from the culture supernatant were determined at 24 h postinfection. The VN^HA,NA^ virus showed comparable infectivity in BMM derived from CLEC5A^−/−^ or WT mice. As observed in the CLEC5A^−^ human M-Mϕ, reduced levels of TNF-α and IP-10 (*P* = 0.063 and *P* = 0.046, respectively) were detected in BMM of CLEC5A^−/−^ mice compared to those in the WT mice after infection (Fig. 6B). Interestingly, a higher level of IFN-α was detected in the CLEC5A^−/−^ mice (*P* < 0.001). The higher induction of the type I interferon response in the BMM derived from the CLEC5A mice was associated with upregulated TLR3 mRNA after influenza virus infection or PIC treatment (Fig. 6C). Overall, BMM derived from CLEC5A^−/−^ mice showed reduced TNF-α and IP-10 but increased IFN-α compared to the WT mice after infection with the VN^HA,NA^ virus or treatment with PIC. The increased IFN-α after influenza infection could be related to the upregulation of TLR3 mRNA in CLEC5A^−/−^ mice, as observed after PIC treatment. The differential type I IFN response in human and murine macrophages suggests that there are different levels of cross talk between CLEC5A-mediated signaling and other cases of PRR-mediated signaling in these two species.

### CLEC5A^−/−^ mice with reduced inflammatory responses but lung viral loads comparable to those of the WT mice were only moderately protected after lethal influenza challenge.

**FIG 7:**
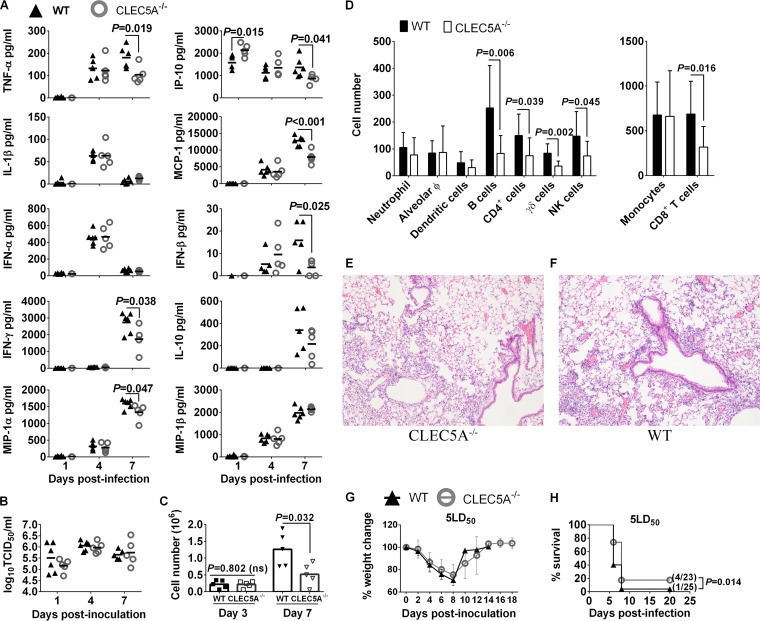
Lower levels of proinflammatory cytokines and reduced level of immune cell infiltration in the lungs of CLEC5A^−/−^ mice compared to the WT mice after lethal challenge of 5 MLD_50_ of VN^HA,NA^ virus. WT and CLEC5A^−/−^ mice were infected with 5 MLD_50_ of VN^HA,NA^ intranasally; mouse lungs were collected at days 1, 4, and 7 postinfection (*n* = 5 or 6 per group at each time point) to determine the concentrations of cytokines and chemokines (means ± SD, in pg/ml) (A) and virus titers (means ± SD, in log_10_ TCID_50_/ml) (B). (C and D) BAL fluid was collected from WT and CLEC5A^−/−^ mice (*n* = 10 per group at each time point) to monitor the total cell count (means ± SD) detected on days 3 and 7 postinfection (C) and the immunophenotyping result on day 7 postinfection using surface markers (*P* values from Mann-Whitney tests are shown) (D). Hematoxylin and eosin staining of the CLEC5A^−/−^ (E) and WT (F) mouse lungs at day 7 postinfection with 5 MLD_50_ of VN^HA,NA^ (*n* = 2 per group). (G and H) Changes in body weight (G) and survival (H) of WT and CLEC5A^−/−^ mice after challenge with 5 MLD_50_ (*P* value from log-rank test is shown).

We further determined the impact of CLEC5A on influenza pathogenesis in CLEC5A^−/−^ and WT mice after lethal challenge with 5 50% murine lethal doses (MLD_50_) (4,766 PFU/mice) of the VN^HA,NA^ virus. On days 1, 4, and 7, mouse lungs were collected to determine the viral titers and levels of cytokines and chemokines. Lower levels of TNF-α, IFN-β, IFN-γ, IP-10, MIP-1α, and MCP-1 were observed in lung homogenates of CLEC5A^−/−^ mice on day 7 postinfection (Fig. 7A) in spite of comparable lung titers in the CLEC5A^−/−^ and WT mice on days 1, 4, and 7 postinfection (Fig. 7B). Corroborated with the proinflammatory cytokine and chemokine levels in lung homogenates, significantly lower numbers of cells were noted in the bronchoalveolar lavage (BAL) fluid collected from the CLEC5A^−/−^ mice compared to the WT mice on day 7 postinfection (Fig. 7C) but not on day 3 postinfection. Specifically, significantly lower numbers of B cells, CD8^+^ T cells, γδ T cells, and NK cells were noted in the CLEC5A^−/−^ mouse lungs (Fig. 7D). The reduced number of CD8^+^ T cells and NK cells correlated with the lower IFN-γ concentration detected in the CLEC5A^−/−^ mouse lungs at day 7 postinfection. Histopathology examination also revealed reduced cell infiltration in the CLEC5A^−/−^ (Fig. 7E) compared to the WT (Fig. 7F) mouse lungs at day 7 postinfection. Despite the differences in lung inflammatory responses on day 7 postinfection, comparable weight loss was observed in both WT and CLEC5A^−/−^ mice (Fig. 7G). A marginally higher survival rate was observed in CLEC5A^−/−^ mice (4/23, 17.4%) compared to wild-type mice (1/25, 4%) (Fig. 7H) (*P* = 0.014 by log-rank test). We observed a more prominent cytokine reduction in the mouse lungs (Fig. 7A) than that observed in BMM (Fig. 6B) derived from CLEC5A^−/−^ and WT mice after influenza infection, possibly due to the mixed cell types present in the mouse lungs.

**FIG 8:**
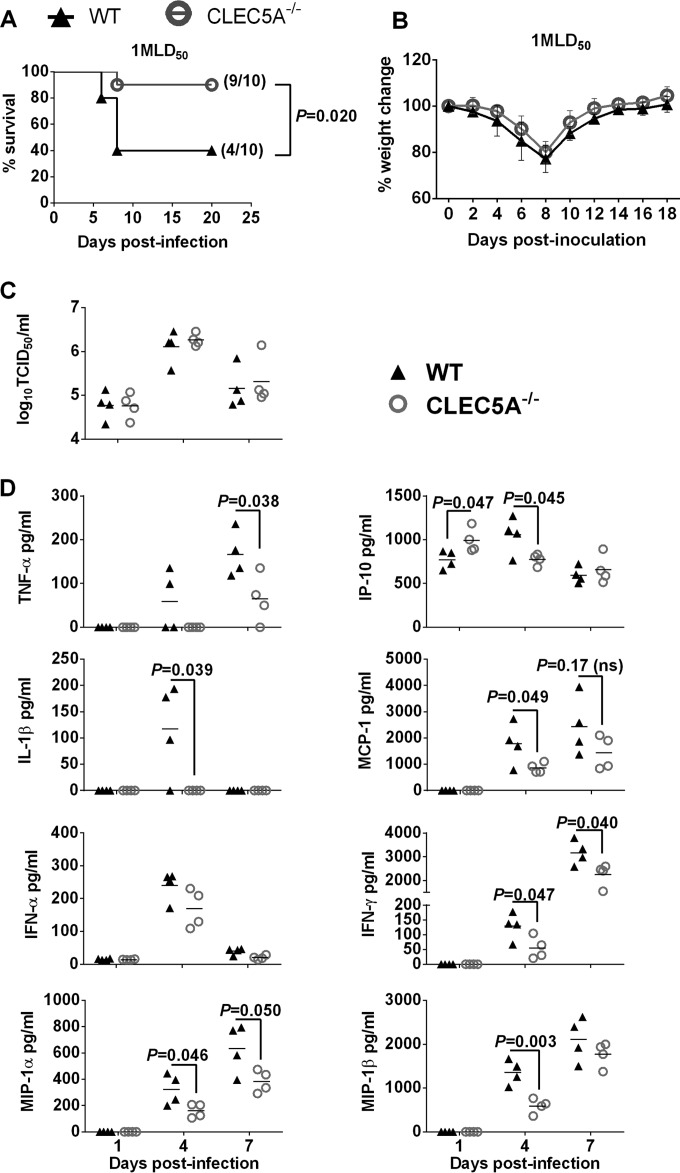
CLEC5A^−/−^ mice were protected from the sublethal challenge of VN^HA,NA^ virus by reducing the level of proinflammatory cytokine in the lungs. WT and CLEC5A^−/−^ mice were infected with 1 MLD_50_ of VN^HA,NA^ intranasally. Mouse survival (A) and body weight changes (B) were monitored every other day; *P* values from log-rank tests are shown. (C and D) Viral titer (means ± SD, in log_10_ TCID_50_/ml) (C) and cytokine and chemokine levels (means ± SD, in pg/ml) (D) detected in the mouse lungs at day 1, 4, and 7 postinfection (*n* = 4 per group at each time point); *P* values from Mann-Whitney tests are shown.

At a lower dose of inoculum (953.2 PFU/mouse, or 1 MLD_50_/mouse), the difference in survival was more prominent, as 90% (9/10) of the CLEC5A^−/−^ mice survived the lethal challenge compared to 40% (4/10) of the matching wild-type mice (Fig. 8A). While there was no difference in mouse weight change (Fig. 8B) or lung viral titers (Fig. 8C), significantly lower proinflammatory cytokine levels were observed in the CLEC5A^−/−^ mouse lungs on day 4 (IP-10, IL-1β, MCP-1, IFN-γ, MIP-1α, and MIP-1β), or day 7 (TNF-α, IFN-γ, and MIP-1α) postinfection (Fig. 8D). Compared to the cytokine levels detected in the CLEC5A^−/−^ and WT mice after infection with 5 MLD_50_ (Fig. 7A), we noted an earlier reduction of the proinflammatory cytokines and chemokines in the CLEC5A^−/−^ mice than the WT mice (Fig. 8D). Since CLEC5A is restricted to myeloid cells, reducing the dose of inoculation allowed us to better assess the potential proinflammatory role of CLEC5A, as inoculation at a higher dose may lead to substantial replication and induction of proinflammatory cytokines in the epithelial cells where the virus replicated equally well in both the CLEC5A^−/−^ and WT mouse lungs. These results also suggest that early reduction of the proinflammatory response is associated with a better survival outcome (as seen with the low-dose challenge). Overall, the data support that CLEC5A mediates induction of proinflammatory cytokine myeloid cells that may lead to differential immune cell infiltration to the lungs and tissue damage.

## DISCUSSION

A series of complex virus-host interactions determine the clinical outcome of influenza infections. Here, we report the identification and validation of CLEC5A as a PRR on myeloid cells that mediates enhanced inflammatory responses after influenza virus infection. In human M-Mϕ or GM-Mϕ, we demonstrated that knocking down CLEC5A expression by gene silencing, blocking influenza-CLEC5A interactions with anti-CLEC5A antibodies, or dampening CLEC5A-mediated signaling using a Syk inhibitor consistently reduced levels of proinflammatory cytokines without affecting the replication of influenza viruses of different subtypes. In murine BMM, reduced IP-10 but elevated IFN-α levels were observed in cells derived from the CLEC5A^−/−^ compared to the WT mice after influenza infection. Upon lethal challenges at 5 MLD_50_ or 1 MLD_50_, CLEC5A^−/−^ mice with reduced inflammatory response and immune cell infiltration in the lungs showed significantly improved survival compared to the WT mice, despite comparable lung viral loads between the CLEC5A^−/−^ and WT mice throughout the course of infections. The survival difference between the WT and CLEC5A^−/−^ mice was more prominent when challenged at a lower lethal dose. Overall, we demonstrated that dampening the CLEC5A-mediated proinflammatory cytokine response in myeloid cells may lower the excessive inflammatory response and improve survival after influenza infections. The results highlight the Syk-coupled CLEC5A signaling pathway as a potential target for immunomodulators to alleviate proinflammatory response associated with influenza immunopathogenesis.

CLEC5A has been demonstrated to mediate inflammatory responses in human or murine macrophages upon DV or JEV infection (26, 37). DV mainly targets monocytes for viral replication, and macrophages are considered a major source of proinflammatory cytokine induction during the course of infection (26). Blocking DV-CLEC5A interaction with intravenous injection of anti-CLEC5A blocking antibodies significantly dampened the serum levels of proinflammatory cytokines in DV2-infected STAT1^−/−^ mice and provided protection against DV2-induced complications, including hemorrhage and plasma leakage, leading to a better survival rate compared to the isotype control antibody-treated mice (26). While influenza can similarly activate CLEC5A-mediated inflammatory responses in the myeloid cells, influenza viruses predominantly replicate at the respiratory epithelium cells. In addition, the mechanisms of influenza virus or flavivirus interaction with CLEC5A may differ, since some of the anti-CLEC5A blocking antibodies previously shown to successfully dampen cytokine induction after DV or JEV infection failed to suppress the secretion of IP-10 in M-Mϕ after influenza virus infection (Fig. 5B). This suggests that the active binding site of CLEC5A for influenza virus is different from that for DV or JEV. The binding sites for the anti-CLEC5A antibodies capable of suppressing cytokine induction after DV, JEV, and influenza virus infection should be further investigated to understand the nature of the ligands for CLEC5A activation.

The cytokine profiles between human PBMC-derived macrophages and murine BMM after influenza infections do not match fully, which could be due to differences in CLEC5A-mediated signaling or differential cross talk with other signaling pathways in these two species. Interspecies differences in the transcriptional response in human and murine macrophages after TLR4-mediated LPS stimulation have been reported (43); in addition, the inducible nitric oxide synthase (*iNOS*) gene can be induced by IFN-γ and LPS stimulation in murine macrophages but not in human macrophages (44). These studies suggest that despite significant evolutionary conservation in the pattern recognition receptors, differences in the downstream gene expression profile between mouse and human can still be observed. We examined available literature aiming to understand the impact of CLEC5A-mediated activation on cytokine production in murine BMMs. Activation of CLEC5A in a murine 32Dcl3 myeloid precursor cell line with agonist antibodies was reported to induce higher mRNA levels of RANTES, IP-10, and CCL22 but not TNF-α (45). This is consistent with our observation that the CLEC5A^−/−^ BMMs secreted significantly lower levels of IP-10 but not TNF-α than the CLEC5A^+^ BMMs after influenza infection. However, we observed a differential IFN-α response triggered in human and murine macrophage after influenza infection, which could be due to differential cross talk between CLEC5A-mediated signaling and other signaling pathways in human and murine macrophages after influenza infection. It is also not known if functional compensation occurs in the CLEC5A knockout mice. Regardless of the differential cytokine profiles exhibited in human macrophages and murine BMM after influenza infections, the proinflammatory role of CLEC5A is consistently shown in both species with different experimental results, including the reduction of proinflammatory cytokines in the macrophages or in the mouse lungs. Further studies are needed to investigate CLEC5A-mediated signaling and the potential cross talk with other signaling pathways in these two species.

Multiple PRRs work in synergy to detect viral RNA or proteins synthesized during influenza replication (46–48). A well-orchestrated host response may help to maintain the inflammatory response while inducing an effective adaptive immune response for viral clearance. We observed survival advantage in CLEC5A^−/−^ mice after lethal influenza challenge, while a more prominent survival advantage was observed at a sublethal dose. This is consistent with a report showing that mice deficient in TLR3 showed variable survival compared to WT mice depending on the dose or strain of influenza virus used for challenge (49, 50). The more prominent survival advantage for the CLEC5A^−/−^ mice over the WT mice at a lower dose of inoculum suggests that in addition to the proinflammatory response elicited in the myeloid cells, viral replication kinetics in the lung epithelium cells and the subsequent release of cytokines from the infected cells also determine the survival outcome. The reduction of proinflammatory cytokine secreted by CLEC5A-expressing monocytes/macrophages recruits lower numbers of NK, B, and T cells to the site of infection, which likely lead to reduced lung damage and eventually increased mouse survival.

Taken together, we identified the Syk-coupled CLEC5A as a PRR for influenza virus that mediates proinflammatory responses in human and murine myeloid cells. Without affecting viral load, dampening CLEC5A-mediated inflammatory responses in myeloid cells provided survival advantage *in vivo* while reducing NK, B, and T cell infiltration into and tissue damage of the mouse lungs. Our results highlight the Syk-coupled CLEC5A signaling pathway as a potential target for immunomodulators to alleviate proinflammatory responses associated with influenza immunopathogenesis.

## MATERIALS AND METHODS

### Generation of pseudoparticles expressing influenza HA.

Pseudoparticles expressing influenza HA were produced as described previously (51). Briefly, the coding sequences of HA derived from VN1203 (H5N1) (PP-VN^HA^) or HK5498 (H1N1) (PP-HK^HA^) were cloned into the pcDNA3.1 vector (Invitrogen) with an 8-amino-acid FLAG peptide (DYKDDDDK) added to the HA C terminus. Lentiviral packaging vector plasmid pNL Luc E^−^ R^−^, which encodes an HIV-1 provirus lacking a functional *env* gene and carries a firefly luciferase reporter gene in place of the *nef* gene (provided by Pierre Charneau, Institute Pasteur, Paris), were cotransfected with pcDNA3.1 with or without coding influenza HA sequence in 293T human embryonic kidney cells using a CalPhos mammalian transfection kit (Clontech). Neuraminidase (5 mU/ml) derived from Vibrio cholerae (Roche) was added to the medium at 36 h posttransfection. The culture medium was harvested at 72 h posttransfection and was further concentrated by sucrose gradient.

### Enzyme-linked immunosorbent assay (ELISA) using soluble human CLR and pseudoparticles expressing influenza HA.

Soluble CLRs were produced as described previously (26). CLRs were diluted using dilution buffer (1% polyvinyl alcohol, 2 mM MgCl_2_, 2 mM CaCl_2_ in Tris-buffered saline) to the concentration of 5 μg/ml and were coated on microtiter plates at 100 μl/well (0.5 μg per well) to capture PP-VN^HA^ or PP-HK^HA^. The bound pseudoparticles were detected by anti-H1 (Abcam) or anti-H5 antibodies (a generous gift from Robert Webster, St. Jude Children's Research Hospital) followed by their respective horseradish peroxidase (HRP)-conjugated secondary antibodies with 3,3′,5,5′-tetramethylbenzidine substrate (Invitrogen).

### Viruses.

Recombinant PR8 expressing HA and NA derived from either VN1203 (VN^HA,NA^) or HK5498 (HK^HA,NA^) was generated as described previously (52). The VN^HA,NA^ virus was a vaccine seed strain, kindly provided by Robert Webster, with its multibasic HA cleavage site replaced by that of a low-pathogenicity avian influenza virus (53). For UV inactivation, influenza viruses were irradiated with UV light (CL-100 ultra violet cross-linker) for 15 min.

### Preparation of human PBMC-derived macrophages and murine bone marrow-derived macrophages.

Human PBMC were separated from buffy coats of healthy donors (Hong Kong Red Cross Blood Transfusion Service) using Ficoll-Paque density gradient (GE Healthcare Life Sciences) under Institutional Review Board (IRB) approval (UW 10-124). Monocytes/macrophages were isolated by plastic adherence and were cultured in complete RPMI medium supplemented with 10% fetal bovine serum (FBS) and 10 ng/ml human M-CSF or GM-CSF (Gibco) for 7 days. Human PBMC-derived dendritic cells were differentiated with 10 ng/ml of IL-4 (Gibco) and 50 ng/ml of GM-CSF for 7 days. The research protocol was approved by the Institutional Review Board of the University of Hong Kong/Hospital Authority Hong Kong West Cluster (UW 10-124). For preparation of murine bone marrow-derived macrophages, bone marrow cells were isolated from femurs and tibias and cultured in RPMI medium supplemented with 10% FBS and 10 ng/ml of mouse M-CSF (R&D Systems) for 7 days.

### CLEC5A gene silencing by siRNA.

SmartPool ON-TARGETplus human CLEC5A siRNA (L-021371; Dharmacon) or ON-TARGETplus nontargeting pool (D-001810; Dharmacon) were transfected into human PBMC-derived macrophages using HiPerFect (Qiagen). Briefly, 100 μM siRNA and 6 μl of HiPerFect reagent (per well) were mixed with RPMI, incubated at room temperature for 15 min, and added to the cells seeded in 24-well plates. Cells were further incubated at 37°C for 72 h before cell sorting. Macrophages transfected with CLEC5A gene-specific siRNAs were sorted using murine anti-human CLEC5A IgG2b antibody (clone 283834) to acquire the CLEC5A^−^ population, while a murine IgG2b isotype control (clone 133303) antibody was applied to yield the CLEC5A^+^ population from the cells transfected with nontargeting control siRNA for comparison.

### Infection and treatment of macrophages.

Differentiated human or murine macrophages were infected with influenza viruses at an MOI of 2. After 1 h of adsorption, the virus inoculum was removed and the cells were washed once with phosphate-buffered saline (PBS), followed by incubation with infection medium (RPMI supplemented with 2% FBS) for 24 h. For Syk inhibition and CLEC5A blocking assay, human PBMC-derived macrophages were pretreated with the indicated concentrations of Bay 61-3606 (Merck Millipore) or anti-human CLEC5A antagonistic antibody for 1 h before and throughout the infection process. Supernatant was collected at 24 h posttreatment to measure the cytokines and chemokines. The 50% cytotoxic concentration (CC_50_) of Bay 61-3606 in M-CSF-differentiated macrophages was 243 μM (data not shown). For treatment with TLR ligands, murine bone marrow-derived macrophages from CLEC5A^−/−^ and WT populations were treated with zymosan (10 μg/ml), poly(I·C) (50 μg/ml), lipopolysaccharide (100 μg/ml), or gardiquimod (10 μg/ml) for 24 h before the supernatant was collected.

### Quantification of cytokines and chemokines.

Culture supernatants collected were irradiated with UV light (CL-100 ultra violet cross-linker) for 15 min to inactivate the viruses. The concentrations of the cytokines and chemokines in the culture supernatant were quantified by FlowCytomix (eBioscience), a multiplex bead-based assay for quantification of multiple cytokines and chemokines using flow cytometers. BD FACSCalibur was used for the experiments, and the concentrations of cytokines and chemokines were determined using FlowCytomix Pro 3.0 software.

### Quantification of viral M gene.

RNA was isolated from the infected cells using an RNeasy minikit (Qiagen, Germany), and cDNA was reverse transcribed by using oligo(dT)_20_ and the SuperScript first-strand synthesis system (Invitrogen). Quantitative real-time PCR analysis was performed using a LightCycler system SW 3.5.3 (Roche). The following influenza M-gene primer sequences were used: forward, 5′-CTTCTAACCGAGGTCGAAACG-3′; reverse, 5′-GGCATTTTGGACAAAGCGTCTA-3′.

### Animal experiments.

The animal experiments were approved by the Committee of the Use of Live Animals for Teaching and Research of the University of Hong Kong (CULATR 3137-13). The CLEC5A^−/−^ population was originally generated by excision of exons 3 to 5 from of the CLEC5A genomic DNA (encoding exons 1 to 7), isolated from a 129/Sv genomic DNA bacterial artificial chromosome library using Cre/loxP-mediated recombination in embryonic stem cells derived from the 129 mouse strain (37), followed by backcrossing 10 generations in a C57BL/6 background before they were interbred at the laboratory of Shie-Liang Hsieh at Academia Sinica, Taiwan. The CLEC5A^−/−^ and the matching C57BL/6 wild-type mice were imported and bred at the Laboratory Animal Unit (AAALAC accredited since 2005) at the University of Hong Kong under the same housing conditions. To monitor viral pathogenesis in CLEC5A^−/−^ and the matching C57BL/6 wild-type mice, mice were anesthetized by intraperitoneal injection of ketamine and xylazine, followed by intranasal inoculation with 4,766 PFU (5 MLD_50_) or 953.2 PFU (1 MLD_50_) of VN^HA.NA^ in 25 μl minimal essential medium (MEM). Mice were monitored daily and clinical symptoms and weight recorded on alternate days. Mice were sacrificed by intraperitoneal injection of pentobarbital on days 1, 4, and 7 postinfection, and the lungs were collected and homogenized in 1 ml of PBS (Omni tissue homogenizer; Omni International). Viral titers were determined in MDCK cells by determining the 50% tissue culture infective dose (TCID_50_), and cytokines or chemokines were quantified by the bead-based cytokine quantification kit (BD Bioscience).

### Flow-cytometric immunophenotyping.

The mouse lungs were lavaged twice with 1 ml of ice-cold RPMI. Red blood cells were lysed, and a small aliquot of the cell suspension was stained with trypan blue, followed by counting of viable cells under a light microscope; the total viable cell count for each BAL fluid sample was calculated accordingly. For immunophenotyping of the cells in the BAL fluid, cells were subjected to Fc receptor blocking (eBioscience) and further stained with antibodies for cell markers (all from BioLegend) for 30 min on ice. Mixture 1 contained fluorescein isothiocyanate (FITC)-CD11c (N418), allophycocyanin (APC)-Cy7-CD11b (M1/70), APC-Ly6G (1A8), phycoerythrin (PE)-Cy7-Ly6C (HK1.4), PE-F4/80 (BM8), and peridinin chlorophyll protein (PerCP)-Cy5.5-MHC-II (I-AE). Mixture 2 contained APC-CD3 (145-2C11), APC-Cy7-CD4 (GK1.5), PerCP-Cy5.5-CD8 (53-6.7), FITC-CD49b (DX5), PE-T cell receptor gamma/delta (TCRγ/δ) (GL3), and PE-Cy7-B220 (RA3-6B2). Cells were classified based on the following surface markers: neutrophils (CD11b^+^ MHC-II^lo^ Ly6C^+^ Ly6G^+^), alveolar Mϕ (F480^+^ MHC-II^hi^ CD11c^+^ Ly6C^+^), inflammatory monocytes (F480^+^ MHC-II^hi^ CD11c^−^ Ly6C^+^), dendritic cells (F480^−^ MHC-II^hi^ CD11b^+^ CD11c^+^ Ly6C^+^), B cells (B220^+^ CD3^−^), CD4^+^ T cells (CD3^+^ CD4^+^ CD8^−^), CD8^+^ T cells (CD3^+^ CD4^−^ CD8^+^), γδ T cells (CD3^+^ TCRγδ^+^), and NK cells (CD49b^+^ CD3^−^). Cells were fixed with 4% paraformaldehyde prior to data acquisition using flow cytometry. The results were analyzed by FlowJo.

### Statistical analysis.

Statistical significance was analyzed using the Mann-Whitney test with a threshold *P* value of <0.05 using GraphPad version 6.0 (GraphPad Software). Survival of mice was compared using log-rank test.

## References

[1] Everitt AR, Clare S, Pertel T, John SP, Wash RS, Smith SE, Chin CR, Feeley EM, Sims JS, Adams DJ, Wise HM, Kane L, Goulding D, Digard P, Anttila V, Baillie JK, Walsh TS, Hume DA, Palotie A, Xue Y, Colonna V, Tyler-Smith C, Dunning J, Gordon SB, GenISIS Investigators, MOSAIC Investigators, Smyth RL, Openshaw PJ, Dougan G, Brass AL, Kellam P 2012 IFITM3 restricts the morbidity and mortality associated with influenza. Nature 484:519–523. doi:10.1038/nature10921. 22446628 PMC3648786

[2] Haller O, Staeheli P, Schwemmle M, Kochs G 2015 Mx GTPases: dynamin-like antiviral machines of innate immunity. Trends Microbiol 23:154–163. doi:10.1016/j.tim.2014.12.003. 25572883

[3] Fukuyama S, Kawaoka Y 2011 The pathogenesis of influenza virus infections: the contributions of virus and host factors. Curr Opin Immunol 23:481–486. doi:10.1016/j.coi.2011.07.016. 21840185 PMC3163725

[4] Kash JC, Taubenberger JK 2015 The role of viral, host, and secondary bacterial factors in influenza pathogenesis. Am J Pathol 185:1528–1536. doi:10.1016/j.ajpath.2014.08.030. 25747532 PMC4450310

[5] Iwasaki A, Pillai PS 2014 Innate immunity to influenza virus infection. Nat Rev Immunol 14:315–328. doi:10.1038/nri3665. 24762827 PMC4104278

[6] Guillot L, Le Goffic R, Bloch S, Escriou N, Akira S, Chignard M, Si-Tahar M 2005 Involvement of toll-like receptor 3 in the immune response of lung epithelial cells to double-stranded RNA and influenza A virus. J Biol Chem 280:5571–5580. doi:10.1074/jbc.M410592200. 15579900

[7] Lund JM, Alexopoulou L, Sato A, Karow M, Adams NC, Gale NW, Iwasaki A, Flavell RA 2004 Recognition of single-stranded RNA viruses by Toll-like receptor 7. Proc Natl Acad Sci U S A 101:5598–5603. doi:10.1073/pnas.0400937101. 15034168 PMC397437

[8] Diebold SS, Kaisho T, Hemmi H, Akira S, Reis e Sousa C 2004 Innate antiviral responses by means of TLR7-mediated recognition of single-stranded RNA. Science 303:1529–1531. doi:10.1126/science.1093616. 14976261

[9] Lee SM, Kok KH, Jaume M, Cheung TK, Yip TF, Lai JC, Guan Y, Webster RG, Jin DY, Peiris JS 2014 Toll-like receptor 10 is involved in induction of innate immune responses to influenza virus infection. Proc Natl Acad Sci U S A 111:3793–3798. doi:10.1073/pnas.1324266111. 24567377 PMC3956146

[10] Pichlmair A, Schulz O, Tan CP, Naslund TI, Liljestrom P, Weber F, Reis e Sousa C 2006 RIG-I-mediated antiviral responses to single-stranded RNA bearing 5′-phosphates. Science 314:997–1001. doi:10.1126/science.1132998. 17038589

[11] Allen IC, Scull MA, Moore CB, Holl EK, McElvania-TeKippe E, Taxman DJ, Guthrie EH, Pickles RJ, Ting JP 2009 The NLRP3 inflammasome mediates in vivo innate immunity to influenza A virus through recognition of viral RNA. Immunity 30:556–565. doi:10.1016/j.immuni.2009.02.005. 19362020 PMC2803103

[12] Ichinohe T, Lee HK, Ogura Y, Flavell R, Iwasaki A 2009 Inflammasome recognition of influenza virus is essential for adaptive immune responses. J Exp Med 206:79–87. doi:10.1084/jem.20081667. 19139171 PMC2626661

[13] Lupfer C, Thomas PG, Anand PK, Vogel P, Milasta S, Martinez J, Huang G, Green M, Kundu M, Chi H, Xavier RJ, Green DR, Lamkanfi M, Dinarello CA, Doherty PC, Kanneganti TD 2013 Receptor interacting protein kinase 2-mediated mitophagy regulates inflammasome activation during virus infection. Nat Immunol 14:480–488. doi:10.1038/ni.2563. 23525089 PMC3631456

[14] Lupfer C, Thomas PG, Kanneganti TD 2014 Nucleotide oligomerization and binding domain 2-dependent dendritic cell activation is necessary for innate immunity and optimal CD8+ T cell responses to influenza A virus infection. J Virol 88:8946–8955. doi:10.1128/JVI.01110-14. 24872587 PMC4136245

[15] de Jong MD, Simmons CP, Thanh TT, Hien VM, Smith GJ, Chau TN, Hoang DM, Chau NV, Khanh TH, Dong VC, Qui PT, Cam BV, Ha Do Q, Guan Y, Peiris JS, Chinh NT, Hien TT, Farrar J 2006 Fatal outcome of human influenza A (H5N1) is associated with high viral load and hypercytokinemia. Nat Med 12:1203–1207. doi:10.1038/nm1477. 16964257 PMC4333202

[16] Cheung CY, Poon LL, Lau AS, Luk W, Lau YL, Shortridge KF, Gordon S, Guan Y, Peiris JS 2002 Induction of proinflammatory cytokines in human macrophages by influenza A (H5N1) viruses: a mechanism for the unusual severity of human disease? Lancet 360:1831–1837. doi:10.1016/S0140-6736(02)11772-7. 12480361

[17] Perrone LA, Plowden JK, Garcia-Sastre A, Katz JM, Tumpey TM 2008 H5N1 and 1918 pandemic influenza virus infection results in early and excessive infiltration of macrophages and neutrophils in the lungs of mice. PLoS Pathog 4:e1000115. doi:10.1371/journal.ppat.1000115. 18670648 PMC2483250

[18] Gao R, Bhatnagar J, Blau DM, Greer P, Rollin DC, Denison AM, Deleon-Carnes M, Shieh WJ, Sambhara S, Tumpey TM, Patel M, Liu L, Paddock C, Drew C, Shu Y, Katz JM, Zaki SR 2013 Cytokine and chemokine profiles in lung tissues from fatal cases of 2009 pandemic influenza A (H1N1): role of the host immune response in pathogenesis. Am J Pathol 183:1258–1268. doi:10.1016/j.ajpath.2013.06.023. 23938324 PMC7119452

[19] Wang Z, Zhang A, Wan Y, Liu X, Qiu C, Xi X, Ren Y, Wang J, Dong Y, Bao M, Li L, Zhou M, Yuan S, Sun J, Zhu Z, Chen L, Li Q, Zhang Z, Zhang X, Lu S, Doherty PC, Kedzierska K, Xu J 2014 Early hypercytokinemia is associated with interferon-induced transmembrane protein-3 dysfunction and predictive of fatal H7N9 infection. Proc Natl Acad Sci U S A 111:769–774. doi:10.1073/pnas.1321748111. 24367104 PMC3896201

[20] Drummond RA, Brown GD 2013 Signalling C-type lectins in antimicrobial immunity. PLoS Pathog 9:e1003417. doi:10.1371/journal.ppat.1003417. 23935480 PMC3723563

[21] Iborra S, Sancho D 2015 Signalling versatility following self and non-self sensing by myeloid C-type lectin receptors. Immunobiology 220:175–184. doi:10.1016/j.imbio.2014.09.013. 25269828 PMC4480263

[22] Sancho D, Reis e Sousa C 2012 Signaling by myeloid C-type lectin receptors in immunity and homeostasis. Annu Rev Immunol 30:491–529. doi:10.1146/annurev-immunol-031210-101352. 22224766 PMC4480235

[23] Herre J, Marshall AS, Caron E, Edwards AD, Williams DL, Schweighoffer E, Tybulewicz V, Reis e Sousa C, Gordon S, Brown GD 2004 Dectin-1 uses novel mechanisms for yeast phagocytosis in macrophages. Blood 104:4038–4045. doi:10.1182/blood-2004-03-1140. 15304394

[24] Leibundgut-Landmann S, Osorio F, Brown GD, Reis e Sousa C 2008 Stimulation of dendritic cells via the dectin-1/Syk pathway allows priming of cytotoxic T-cell responses. Blood 112:4971–4980. doi:10.1182/blood-2008-05-158469. 18818389

[25] Graham LM, Gupta V, Schafer G, Reid DM, Kimberg M, Dennehy KM, Hornsell WG, Guler R, Campanero-Rhodes MA, Palma AS, Feizi T, Kim SK, Sobieszczuk P, Willment JA, Brown GD 2012 The C-type lectin receptor CLECSF8 (CLEC4D) is expressed by myeloid cells and triggers cellular activation through Syk kinase. J Biol Chem 287:25964–25974. doi:10.1074/jbc.M112.384164. 22689578 PMC3406680

[26] Chen ST, Lin YL, Huang MT, Wu MF, Cheng SC, Lei HY, Lee CK, Chiou TW, Wong CH, Hsieh SL 2008 CLEC5A is critical for dengue-virus-induced lethal disease. Nature 453:672–676. doi:10.1038/nature07013. 18496526

[27] Wu MF, Chen ST, Yang AH, Lin WW, Lin YL, Chen NJ, Tsai IS, Li L, Hsieh SL 2013 CLEC5A is critical for dengue virus-induced inflammasome activation in human macrophages. Blood 121:95–106. doi:10.1182/blood-2012-05-430090. 23152543

[28] Ling MT, Tu W, Han Y, Mao H, Chong WP, Guan J, Liu M, Lam KT, Law HK, Peiris JS, Takahashi K, Lau YL 2012 Mannose-binding lectin contributes to deleterious inflammatory response in pandemic H1N1 and avian H9N2 infection. J Infect Dis 205:44–53. doi:10.1093/infdis/jir691. 22080095 PMC3242741

[29] Hartshorn KL, Crouch EC, White MR, Eggleton P, Tauber AI, Chang D, Sastry K 1994 Evidence for a protective role of pulmonary surfactant protein D (SP-D) against influenza A viruses. J Clin Investig 94:311–319. doi:10.1172/JCI117323. 8040272 PMC296311

[30] Hansen S, Selman L, Palaniyar N, Ziegler K, Brandt J, Kliem A, Jonasson M, Skjoedt MO, Nielsen O, Hartshorn K, Jorgensen TJ, Skjodt K, Holmskov U 2010 Collectin 11 (CL-11, CL-K1) is a MASP-1/3-associated plasma collectin with microbial-binding activity. J Immunol 185:6096–6104. doi:10.4049/jimmunol.1002185. 20956340

[31] Yang ML, Chen YH, Wang SW, Huang YJ, Leu CH, Yeh NC, Chu CY, Lin CC, Shieh GS, Chen YL, Wang JR, Wang CH, Wu CL, Shiau AL 2011 Galectin-1 binds to influenza virus and ameliorates influenza virus pathogenesis. J Virol 85:10010–10020. doi:10.1128/JVI.00301-11. 21795357 PMC3196456

[32] Andersen O, Vilsgaard Ravn K, Juul Sorensen I, Jonson G, Holm Nielsen E, Svehag SE 1997 Serum amyloid P component binds to influenza A virus haemagglutinin and inhibits the virus infection in vitro. Scand J Immunol 46:331–337. doi:10.1046/j.1365-3083.1997.d01-147.x. 9350282

[33] Londrigan SL, Turville SG, Tate MD, Deng YM, Brooks AG, Reading PC 2011 N-linked glycosylation facilitates sialic acid-independent attachment and entry of influenza A viruses into cells expressing DC-SIGN or L-SIGN. J Virol 85:2990–3000. doi:10.1128/JVI.01705-10. 21191006 PMC3067946

[34] Ng WC, Liong S, Tate MD, Irimura T, Denda-Nagai K, Brooks AG, Londrigan SL, Reading PC 2014 The macrophage galactose-type lectin can function as an attachment and entry receptor for influenza virus. J Virol 88:1659–1672. doi:10.1128/JVI.02014-13. 24257596 PMC3911607

[35] Reading PC, Miller JL, Anders EM 2000 Involvement of the mannose receptor in infection of macrophages by influenza virus. J Virol 74:5190–5197. doi:10.1128/JVI.74.11.5190-5197.2000. 10799594 PMC110872

[36] Upham JP, Pickett D, Irimura T, Anders EM, Reading PC 2010 Macrophage receptors for influenza A virus: role of the macrophage galactose-type lectin and mannose receptor in viral entry. J Virol 84:3730–3737. doi:10.1128/JVI.02148-09. 20106926 PMC2849513

[37] Chen ST, Liu RS, Wu MF, Lin YL, Chen SY, Tan DT, Chou TY, Tsai IS, Li L, Hsieh SL 2012 CLEC5A regulates Japanese encephalitis virus-induced neuroinflammation and lethality. PLoS Pathog 8:e1002655. doi:10.1371/journal.ppat.1002655. 22536153 PMC3334897

[38] Mok KP, Wong CH, Cheung CY, Chan MC, Lee SM, Nicholls JM, Guan Y, Peiris JS 2009 Viral genetic determinants of H5N1 influenza viruses that contribute to cytokine dysregulation. J Infect Dis 200:1104–1112. doi:10.1086/605606. 19694514 PMC4028720

[39] Hui KP, Lee SM, Cheung CY, Ng IH, Poon LL, Guan Y, Ip NY, Lau AS, Peiris JS 2009 Induction of proinflammatory cytokines in primary human macrophages by influenza A virus (H5N1) is selectively regulated by IFN regulatory factor 3 and p38 MAPK. J Immunol 182:1088–1098. doi:10.4049/jimmunol.182.2.1088. 19124752

[40] Bakker AB, Baker E, Sutherland GR, Phillips JH, Lanier LL 1999 Myeloid DAP12-associating lectin (MDL)-1 is a cell surface receptor involved in the activation of myeloid cells. Proc Natl Acad Sci U S A 96:9792–9796. doi:10.1073/pnas.96.17.9792. 10449773 PMC22289

[41] Cheung R, Shen F, Phillips JH, McGeachy MJ, Cua DJ, Heyworth PG, Pierce RH 2011 Activation of MDL-1 (CLEC5A) on immature myeloid cells triggers lethal shock in mice. J Clin Investig 121:4446–4461. doi:10.1172/JCI57682. 22005300 PMC3204838

[42] Yamamoto N, Takeshita K, Shichijo M, Kokubo T, Sato M, Nakashima K, Ishimori M, Nagai H, Li YF, Yura T, Bacon KB 2003 The orally available spleen tyrosine kinase inhibitor 2-[7-(3,4-dimethoxyphenyl)-imidazo[1,2-c]pyrimidin-5-ylamino]nicotinamide dihydrochloride (BAY 61-3606) blocks antigen-induced airway inflammation in rodents. J Pharmacol Exp Ther 306:1174–1181. doi:10.1124/jpet.103.052316. 12766258

[43] Schroder K, Irvine KM, Taylor MS, Bokil NJ, Le Cao KA, Masterman KA, Labzin LI, Semple CA, Kapetanovic R, Fairbairn L, Akalin A, Faulkner GJ, Baillie JK, Gongora M, Daub CO, Kawaji H, McLachlan GJ, Goldman N, Grimmond SM, Carninci P, Suzuki H, Hayashizaki Y, Lenhard B, Hume DA, Sweet MJ 2012 Conservation and divergence in Toll-like receptor 4-regulated gene expression in primary human versus mouse macrophages. Proc Natl Acad Sci U S A 109:E944–E953. doi:10.1073/pnas.1110156109. 22451944 PMC3341041

[44] Schneemann M, Schoedon G, Hofer S, Blau N, Guerrero L, Schaffner A 1993 Nitric oxide synthase is not a constituent of the antimicrobial armature of human mononuclear phagocytes. J Infect Dis 167:1358–1363. doi:10.1093/infdis/167.6.1358. 7684756

[45] Aoki N, Kimura Y, Kimura S, Nagato T, Azumi M, Kobayashi H, Sato K, Tateno M 2009 Expression and functional role of MDL-1 (CLEC5A) in mouse myeloid lineage cells. J Leukoc Biol 85:508–517. 19074552 10.1189/jlb.0508329

[46] Ichinohe T, Pang IK, Iwasaki A 2010 Influenza virus activates inflammasomes via its intracellular M2 ion channel. Nat Immunol 11:404–410. doi:10.1038/ni.1861. 20383149 PMC2857582

[47] Ramirez-Ortiz ZG, Prasad A, Griffith JW, Pendergraft WF III, Cowley GS, Root DE, Tai M, Luster AD, El Khoury J, Hacohen N, Means TK 2015 The receptor TREML4 amplifies TLR7-mediated signaling during antiviral responses and autoimmunity. Nat Immunol 16:495–504. doi:10.1038/ni.3143. 25848864 PMC4406861

[48] Pothlichet J, Meunier I, Davis BK, Ting JP, Skamene E, von Messling V, Vidal SM 2013 Type I IFN triggers RIG-I/TLR3/NLRP3-dependent inflammasome activation in influenza A virus infected cells. PLoS Pathog 9:e1003256. doi:10.1371/journal.ppat.1003256. 23592984 PMC3623797

[49] Leung YH, Nicholls JM, Ho CK, Sia SF, Mok CK, Valkenburg SA, Cheung P, Hui KP, Chan RW, Guan Y, Akira S, Peiris JS 2014 Highly pathogenic avian influenza A H5N1 and pandemic H1N1 virus infections have different phenotypes in Toll-like receptor 3 knockout mice. J Gen Virol 95:1870–1879. doi:10.1099/vir.0.066258-0. 24878639 PMC4135086

[50] Le Goffic R, Balloy V, Lagranderie M, Alexopoulou L, Escriou N, Flavell R, Chignard M, Si-Tahar M 2006 Detrimental contribution of the Toll-like receptor (TLR)3 to influenza A virus-induced acute pneumonia. PLoS Pathog 2:e53. doi:10.1371/journal.ppat.0020053. 16789835 PMC1475659

[51] Nefkens I, Garcia JM, Ling CS, Lagarde N, Nicholls J, Tang DJ, Peiris M, Buchy P, Altmeyer R 2007 Hemagglutinin pseudotyped lentiviral particles: characterization of a new method for avian H5N1 influenza sero-diagnosis. J Clin Virol 39:27–33. doi:10.1016/j.jcv.2007.02.005. 17409017

[52] Hoffmann E, Neumann G, Kawaoka Y, Hobom G, Webster RG 2000 A DNA transfection system for generation of influenza A virus from eight plasmids. Proc Natl Acad Sci U S A 97:6108–6113. doi:10.1073/pnas.100133697. 10801978 PMC18566

[53] Li S, Liu C, Klimov A, Subbarao K, Perdue ML, Mo D, Ji Y, Woods L, Hietala S, Bryant M 1999 Recombinant influenza A virus vaccines for the pathogenic human A/Hong Kong/97 (H5N1) viruses. J Infect Dis 179:1132–1138. doi:10.1086/314713. 10191214

